# Collagen-induced arthritis and imiquimod-induced psoriasis develop independently of interleukin-33

**DOI:** 10.1186/s13075-016-1042-x

**Published:** 2016-06-18

**Authors:** Sara Khaleghparast Athari, Elodie Poirier, Jérôme Biton, Luca Semerano, Roxane Hervé, Aurélie Raffaillac, Delphine Lemeiter, André Herbelin, Jean-Philippe Girard, Frédéric Caux, Marie-Christophe Boissier, Natacha Bessis

**Affiliations:** INSERM, UMR 1125, 93017 Bobigny, France; Sorbonne Paris Cité Université Paris 13, 74 rue Marcel Cachin, 93000 Bobigny, France; Present address: INSERM UMRS 1138 Equipe 13, Centre de Recherche des Cordeliers, 75006 Paris, France; INSERM U1082, Pôle Biologie Santé, CHU Poitiers, BP 633, Poitiers, 86022 France; Assistance Publique-Hôpitaux de Paris, Avicenne Hospital, Rheumatology Department, 93009 Bobigny, France; Institut de Pharmacologie et de Biologie Structurale (IPBS) CNRS-Université de Toulouse III, 31077 Toulouse, France; Assistance Publique-Hôpitaux de Paris, Avicenne Hospital, Dermatology Department, 93009 Bobigny, France

**Keywords:** IL-33, Arthritis, Psoriasis, T cells

## Abstract

**Background:**

Interleukin (IL)-33 is a dual cytokine with both an alarmin role and a T helper 2 cell (Th2)-like inducing effect. It is involved in the pathogenesis of rheumatoid arthritis (RA) and its models; we recently demonstrated that exogenous IL-33 could inhibit collagen-induced arthritis (CIA) in C57BL/6 mice. However, its pathophysiological role in RA is unclear. Indeed, mice deficient in the IL-33 receptor ST2 show reduced susceptibility to arthritis, and the disease is not modified in IL-33-deficient mice. We examined the immune response in wild-type (WT) and IL-33-deficient mice with CIA. To further understand the role of endogenous IL-33 in inflammatory diseases, we studied its role in a skin psoriasis model. Mice on a C57BL/6 background were deficient in *IL-33* but expressed lacZ under the IL-33 promoter. Therefore, IL-33 promotor activity could be analyzed by lacZ detection and IL-33 gene expression was analyzed by X-Gal staining in various mice compartments. Frequencies of CD4^+^FoxP3^+^ regulatory T cells (Tregs) and Th1 and Th17 cells were evaluated by flow cytometry in WT and IL-33^-/-^ mice. Bone resorption was studied by evaluating osteoclast activity on a synthetic mineral matrix. Psoriasis-like dermatitis was induced by application of imiquimod to the skin of mice.

**Results:**

Severity of CIA was similar in IL-33^-/-^ and WT littermates. Joints of IL-33^-/-^ mice with CIA showed IL-33 promotor activity. In mice with CIA, frequencies of Tregs, Th1 and Th17 in the spleen or lymph nodes did not differ between the genotypes; osteoclast activity was higher but not significantly in IL-33^-/-^ than WT mice. Psoriasis development did not differ between the genotypes.

**Conclusions:**

Despite its expression in the synovium of arthritic mice and normal keratinocytes, IL-33 is not required for CIA development in arthritis or psoriasis. Its absence does not induce a T cell shift toward Th1, Th17 or Treg subpopulations. Altogether, these data and our previous ones, showing that exogenous IL-33 can almost completely inhibit CIA development, suggest that this cytokine is not crucial for development of chronic inflammation. Studies of RA patients are needed to determine whether treatment targeting the IL-33/ST2 axis would be effective.

## Background

Interleukin (IL)-33 is a member of the IL-1 family, which also includes IL-1α, IL-1β, IL-18, IL-36, IL-37, IL-38, and the receptor antagonists IL-1Ra and IL-36Ra [1]. It is localized in the cell nucleus [2] and was first described as a cytokine passively released by necrotic epithelial and endothelial cells with tissue damage during infection or inflammatory diseases, thereby acting as an alarmin. However, IL-33-expressing cells also include other cells of stromal and parenchymal origin, such as macrophages, dendritic cells, mast cells, smooth muscle cells, fibroblasts, glial cells, osteoblasts, synoviocytes, and adipocytes.

IL-33 signals via a heteromeric receptor consisting of ST2 and IL-1R accessory protein (IL-1RAcP). Several activated immune cells and numerous nonimmune cells respond to IL-33 via its receptor. Immune cells expressing IL-33 receptor include T helper 2 (Th2) cells (but not Th1 and Th17), all polymorphonuclear neutrophils, induced natural killer T cells, natural killer cells, B lymphocytes, macrophages, dendritic cells, and type 2 innate lymphoid cells (ILC2) [3]. The expression pattern of IL-33 receptor indicates that it is in the center of a complex cellular network of immune and nonimmune cells [4].

IL-33 is considered to be a dual-role cytokine that can both promote and reduce inflammation depending on the tissue and cytokine environment. Its positive or negative control of inflammation involves a complex process. Its pro-inflammatory role is related to the involvement of the IL-33/ST2 system in regulating each phase of a generic inflammatory pathway. Indeed, when tissues receive insults leading to inflammation, the major sources of IL-33 are epithelial cells, endothelial cells, or tissue macrophages. IL-33 then induces the production of additional inflammatory mediators and amplifies the inflammatory response. The anti-inflammatory role of IL-33 is related to its capacity to suppress inflammation by driving type 2 immunity. IL-33 acts by promoting M2 macrophage [5] or Th2 T cell differentiation. IL-33 can also expand and induce high levels of IL-5 and IL-13 production by ILC2.

Some works suggest a functional role of the IL-33/ST2 axis in the pathogenesis of human and mouse arthritis. In rheumatoid arthritis (RA) patients, IL-33 levels are elevated in serum and synovial fluid [6, 7] and strong IL-33 expression can be detected in endothelial cells and fibroblasts in the human RA synovium [8]. IL-33 induces in vitro tumor necrosis factor (TNF) alpha and IL-8 production by human mast cells within the synovium [9]. In mouse models of experimental arthritis induced by active immunization, such as collagen- and antigen-induced arthritis, the use of ST2-knockout (KO) mice, ST2 blockade, or injection of soluble ST2 led to decreased immune responses and severity of arthritis, which suggested a pathogenic role for IL-33 signaling through ST2 in these experimental models [10, 11]. However, K/BxN serum transfer-induced arthritis is reduced in ST2-knockout mice but does not differ in IL-33-knockout and wild-type (WT) mice [12]. As well, sST2, the soluble receptor for IL-33, has no effect on collagen-induced arthritis (CIA) [13]. IL-33 deficiency did not modify antigen-induced arthritis or CIA in DBA/1 mice [14].

As RA, psoriasis is a chronic inflammatory disease, and IL-33 may have a role in its pathogenesis. Indeed, IL-33 level is increased in skin lesions of patients with psoriasis [15] and is decreased after anti-TNF-α treatment [16]. Its role as an alarmin is suggested by the positive Köbner reaction, described as new psoriasis lesions after slight skin injury. The imiquimod (IMQ)-induced psoriasiform dermatitis acts via Toll-like receptor 7 (TLR7) stimulation with daily application of Aldara® cream, which contains IMQ [17].

These studies in arthritis models underscore the complex nature of IL-33 and that it may also play a role in psoriasis. Therefore, we used in vitro models to investigate whether endogenous IL-33 administration can contribute to the regulatory and inflammatory pathways culminating in the pathology of chronic inflammatory diseases.

## Methods

### Mice

IL-33^-/-^ mice were a gift from JP Girard and generated as described [18]. In these mice, a β-geo insertion disrupts production of the IL-33 protein, which is useful to visualize the activity of the endogenous IL-33 promoter via X-Gal staining (β-galactosidase activity). Heterozygous IL-33^+/-^ mice were generated by breeding C57BL/6 mice (Janvier Labs, Le Genest-Saint-Isle, France) to IL-33^-/-^ mice. Then IL-33^+/-^ mice were bred together and the resulting homozygous IL-33^+/+^ WT littermates were used as controls in all experiments.

### Collagen-induced arthritis (CIA) induction and evaluation

Arthritis was induced with native chicken collagen type II (CII, MD Biosciences, Zürich, Switzerland). At 10 to 12 weeks of age, male C57BL/6 mice were injected intradermally at the base of the tail with 50 μg CII emulsified in complete Freund’s adjuvant (CFA, MD Biosciences). On day 21, mice received a booster subcutaneous injection of CII emulsified in CFA. A blinded procedure was used to monitor clinical and histological arthritis in all four limbs, as previously described [19, 20].

### Induction and evaluation of IMQ-induced psoriasis-like dermatitis

Psoriasis was induced in mice with commercially available IMQ cream (5 %; Aldara cream; MEDA AB, Solna, Sweden). Male mice, 9 to 11 weeks old, received a daily topical application of 62.5 mg IMQ cream on the shaved back skin for 10 consecutive days. A blinded procedure was used to monitor psoriasis-like skin inflammation. Clinical scoring of psoriasis based on the human clinical Psoriasis Area and Severity Index (PASI) was performed daily. Three variables were evaluated – erythema, scaling, and thickening – scored independently on a scale from 0 to 4: 0, none; 1, slight; 2, moderate; 3, marked; 4, very marked. The cumulative score was a total of the three parameter scores ranging from 0 to 12. For histological assessment, skin samples from the back were fixed in 10 % formalin for at least 24 h at room temperature and embedded in paraffin. Deparaffinized sections of 4 μm were stained with hematoxylin erythrosine saffron before microscopy observation (optical microscopy). Epidermal thickness was evaluated by using Archimed software (Microvision Instruments, Les Ulis, France). We averaged five to ten values for independent fields of epidermis (from corneal layer to the basement membrane zone) for each mouse.

### β-galactosidase detection

Hind paws were dissected and fixed in a solution of glutaraldehyde 25 % for 2 to 4 h at room temperature. Knee joints were injected with the chromogenic substrate X-Gal (from the lacZ tissue staining kit, InvivoGene, Toulouse, France) and incubated in the solution for 3 h at 37 °C under shaking. The paws were washed overnight in phosphate-buffered saline (PBS) 1X (37 °C). Sections of 7 μm were prepared and stained with hematoxylin and eosin (H&E) before microscopy observation.

Skin samples were dissected and stored at -80° until use. Skin cryosections of 5 μm were embedded in OCT, then fixed for 10 min in PBS with 0.2 % glutaraldhehyde and rinsed three times in washing buffer (MgCl_2_ 2 mM, Na desoxycholate 0.01 %, Igepal 0.02 %, PBS) for 5 min. For ß-gal staining, slices were incubated overnight with X-gal (1 mg/ml) in staining buffer (potassium ferricyanide 6 mM, potassium ferrocyanide 6 mM), at 37 °C. Sections were then rinsed in PBS, distilled water and counterstained with eosin. Blue color was detected by light microscopy.

### IL-33 detection by immunofluorescence

Paraffin sections (5 μm) were deparaffinized in a Zylene mix of isomer solution (Carlo Erba Reagents, Val-de-Reuil, France) and rehydrated in a graded alcohol series. Then the sections were washed in distilled water and boiled in a microwave oven for epitope retrieval in sodium citrate buffer (10 mM, pH 6). Slides were incubated with polyclonal goat immunoglobulin (Ig)G anti-mouse IL-33 (Clone: Q8BVZ5, 1:200, R&D Systems, Minneapolis, MN, USA) or control isotype (polyclonal goat IgG, 1:200 R&D Systems), then washed in PBS (5 min, three times) and incubated with donkey IgG anti-goat IgG conjugated with Northern Lights 557 (1:200 R&D Systems) in darkness. Slides were counterstained with DAPI.

### Osteoclastogenesis and bone resorption

For osteoclastogenesis assay, the anterior paws were dissected to collect bone marrow cells. Then, for each mouse, 2.10^5^ cells were plated in triplicate on 96-well plates with receptor activator of nuclear factor kappa B ligand (RANKL) (100 ng/mL) and macrophage colony-stimulating factor (M-CSF) (30 ng/mL) in 5 % CO_2_ at 37 °C. Cells were stained with TRAP (Acid Phosphatase, Leukocyte TRAP Kit, Sigma-Aldrich, St Louis, MO, USA) on day 5, and osteoclasts with ≥3 nuclei were identified as TRAP-positive.

For bone resorption assay, bone marrow cells were isolated as described above. Then, 4.10^5^ cells were cultured with mouse M-CSF (30 ng/ml) and mouse RANKL (100 ng/ml) on an Osteo Assay plate (Corning Inc., Corning, NY, USA) containing a synthetic mineral matrix with 5 % CO_2_ at 37 °C. At day 8, bone resorption was analyzed. The experiment was performed in triplicate wells for each mouse and one image was obtained from one whole well. Number of resorption spots and percentage resorption area were evaluated by using ImageJ, and mean number from each triplicate experiment was calculated.

### Cell preparation and analysis

Leukocytes from the spleen were prepared by using a cell strainer, and red blood cells were lysed in hemolytic buffer (NH_4_Cl, KHCO_3_, and EDTA). Afferent and popliteal lymph nodes were dissected out of the hind limbs and leukocytes were prepared using a homogenizer. Blood was collected by heart puncture, and for cell analysis, red blood cells were lysed in hemolytic buffer. Leukocytes were stained with PercPCyc5.5-anti CD4 antibody (clone RM4-5) (BD Biosciences). The APC-anti-Foxp3 Staining Set (clone FJK-16 s) (eBioscience, San Diego, CA, USA) was used for intracellular staining. To analyze Th1 and Th17 cells, 10^6^ leukocytes were stimulated with PMA (50 ng/ml) and ionomycin (1 μg/ml) (Sigma-Aldrich) with monensin (BD Biosciences) for 4 h. For intracellular interferon (IFN)-γ and IL-17 staining, CD4^+^ cells were first stained with a surface marker and permeabilized with Cytofix/Cytoperm (BD Biosciences), then stained with the antibodies APC-anti-IFN-γ (clone XMG1.2) or PE-anti-IL-17 (clone TC11-18H10) (BD Biosciences). Flow cytometry involved use of FACS Calibur (Becton Dickinson, Mountain View, CA, USA). Dead cells were excluded by forward and side scatter characteristics (BD Biosciences) and results were analyzed by using CellQuest Pro (BD Biosciences).

## Results

### Endogenous IL-33 is expressed in mouse joints

**Fig. 1:**
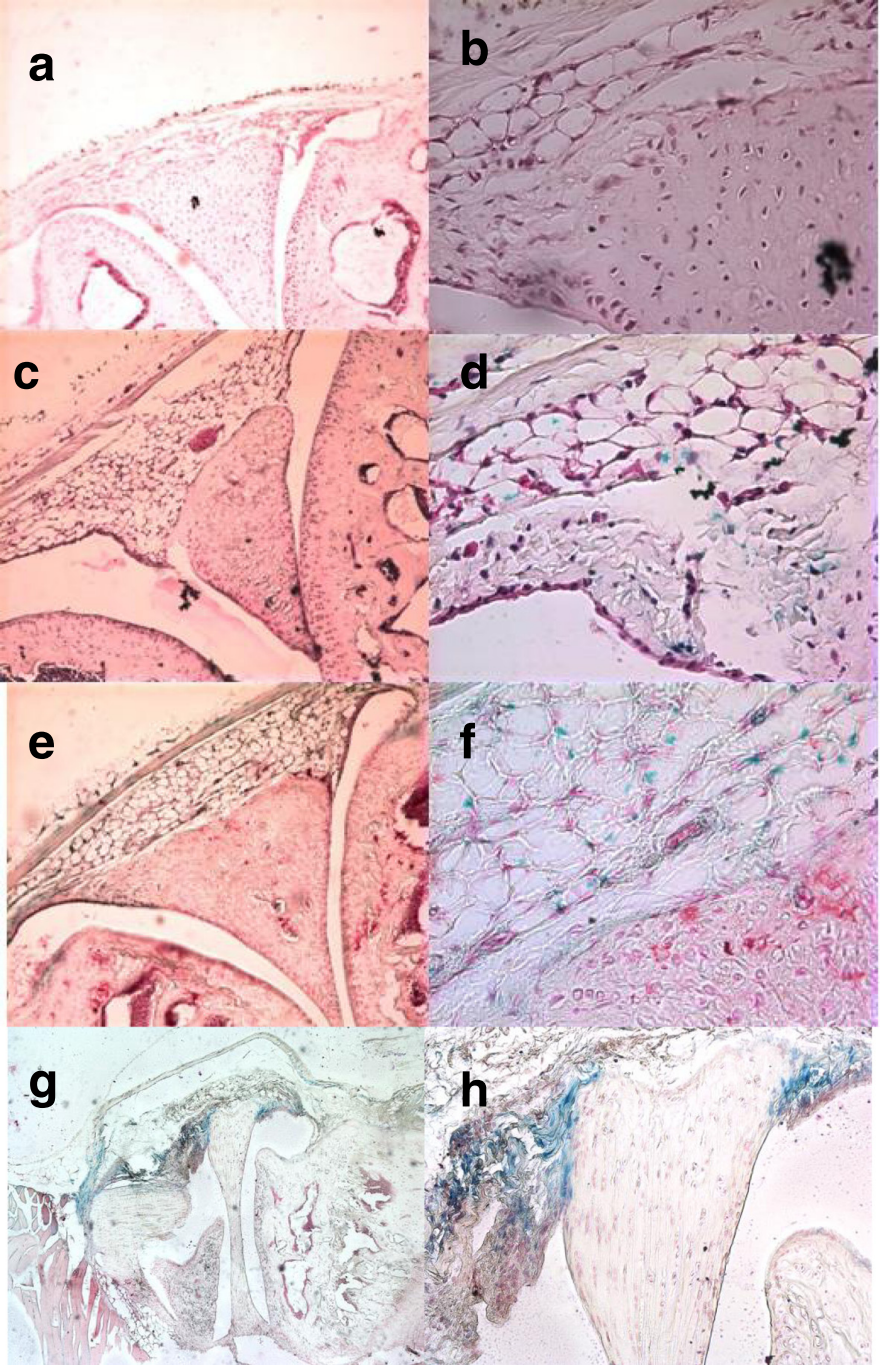
LacZ expression in mouse joints with or without collagen-induced arthritis (CIA). Knee joint sections of mice without CIA (**a**, **b**, **c**, **d**) or with CIA (day 10 (**e**, **f**) or day 35 (**g**, **h**) after CIA induction were stained with X-gal, and blue staining was observed at × 10 (a, c, e, g) or × 20 (h) or × 40 (b, d, f) magnification. IL-33+/+ wild-type (WT) mice (a, b) and interleukin (IL)-33^-/-^ mice (c, d, e, f, g, h) were analyzed. Slides are representative of eight WT mice, eight IL-33^-/-^ mice without CIA, five IL-33^-/-^ mice at day 10 after CIA, and four IL-33^-/-^ mice at day 35 after CIA

X-Gal staining of knee joints from IL-33^-/-^ or WT IL-33^+/+^ mice was used to evaluate IL-33 promotor activity in mice with or without arthritis. As expected, no β-galactosidase activity was observed in joints from WT mice, without the IL-33–LacZ construction (Fig. 1a and b). In contrast, LacZ expression was detected in IL-33^-/-^ mice without CIA, so the IL-33 promoter was constitutively active in naive mice (Fig. 1c and d). We then analyzed the expression of IL-33 in mice with CIA at day 10 or 35 and found LacZ expression in joint tissues (Fig. 1e–h).

### Collagen-induced arthritis is not affected by lack of IL-33

**Fig. 2:**
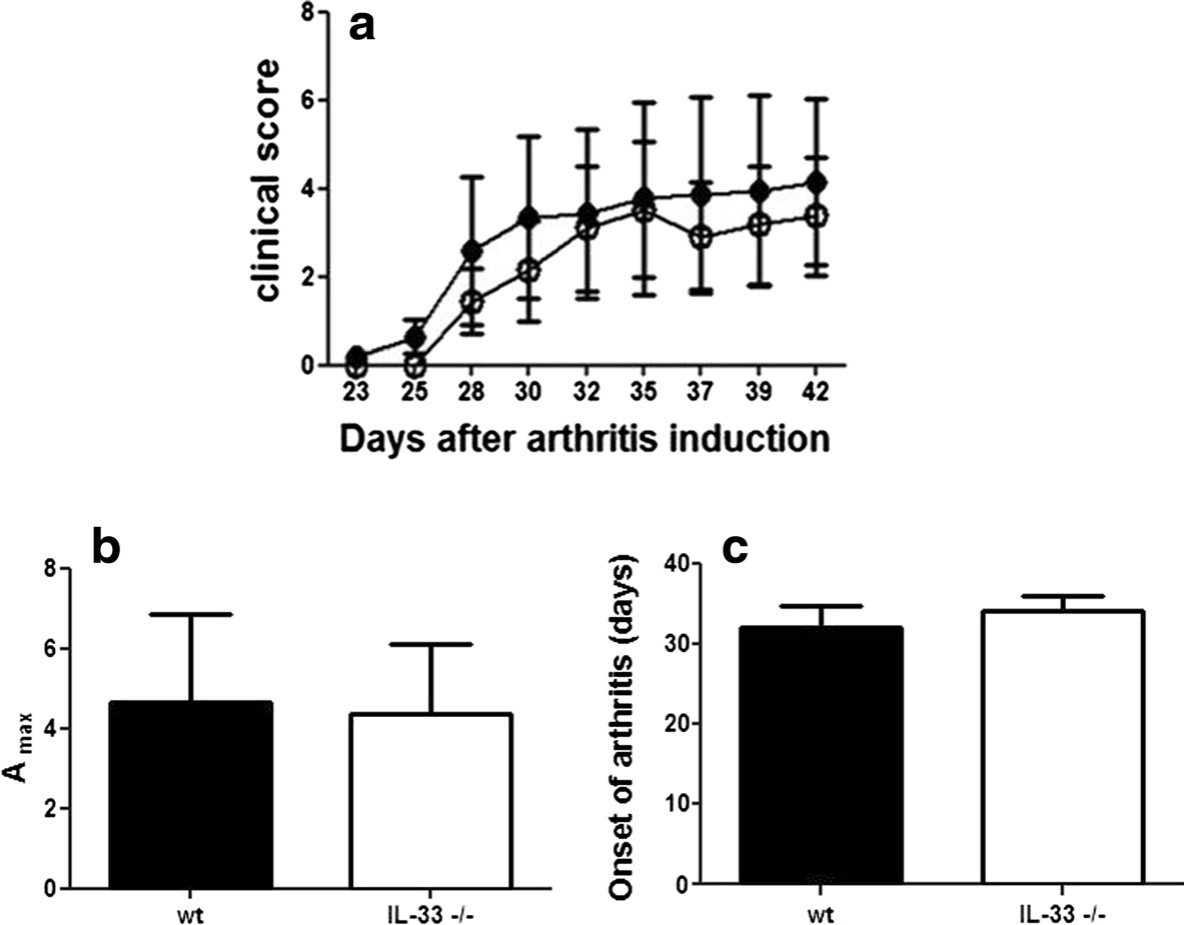
IL-33 deficiency does not affect collagen-induced arthritis. IL-33^-/-^ mice (*n* = 13) *open circles* and WT mice (*n* = 12, *closed circles*) were injected intradermally with chicken collagen II emulsified in complete Freund’s adjuvant (CFA) on days 0 and 21. **a** Clinical arthritis scores. **b** Mean maximal arthritis score (A_max_). **c** Mean onset of arthritis. Data are mean ± SEM and are representative of one experiment in two similar experiments *IL* interleukin, *WT* wild-type

We then investigated the effect of IL-33 deficiency on the development of CIA. Clinical scoring revealed that the course of arthritis was similar in IL-33^-/-^ and WT mice (Fig. 2a). Moreover, the mean maximal arthritis score (A_max_) and onset of arthritis did not differ in IL-33^-/-^ and WT mice (Fig. 2b and c). Finally, histological examination at day 45 revealed no difference between IL-33^-/-^ and WT mice in joint inflammation score (0.71 ± 0.23 vs. 0.74 ± 0.18) and destruction score (0.38 ± 0.16 vs. 0.38 ± 0.13).

### Effect of endogenous IL-33 on T-cell subset frequencies during CIA

**Fig. 3:**
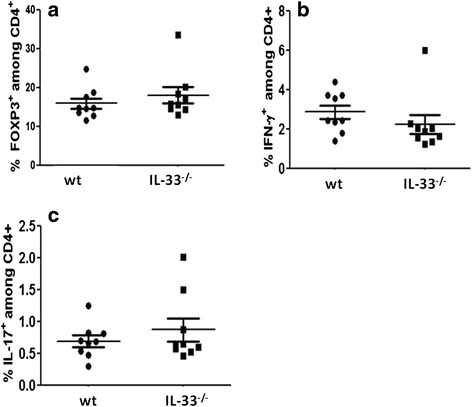
Effect of IL-33 deficiency on T helper (Th) cell subsets. Mice as in Fig. 2 were killed 45 days after the first injection. Splenocytes from nine mice randomly selected in both groups were stained with fluorochrome-conjugated anti-CD4, anti-FoxP3, anti-IFN-γ and anti-IL-17 antibodies. Regulatory T cells (Tregs) were defined as CD4^+^ FoxP3^+^ cells, and Th1 and Th17 cells were defined as CD4^+^ IFN-γ^+^ and CD4^+^ IL-17^+^ cells, respectively. The proportion of FoxP3^+^ (**a**), IFN-γ^+^ (**b**), and IL-17^+^ cells (**c**) among CD4^+^ cells are shown. Data are representative of one experiment in two similar experiments. *IFN* interferon, *IL* interleukin, *WT* wild-type

Because IL-33 was found to induce a type 2 immune response [21–23] and regulatory T cell (Treg) expansion [24], we wondered whether, despite a lack of IL-33 deficiency effect on CIA, the lack of this cytokine could lead to a modified T cell profile in CIA. Therefore, we evaluated the frequencies of CD4+ T cell subpopulations at 45 days after CIA induction in WT and IL-33^-/-^ mice. Frequencies of regulatory T cells defined as CD4^+^FoxP3^+^ cells and Th1 or Th17 cells in the spleen (and lymph nodes, data not shown) did not differ between IL-33^-/-^ and WT mice during CIA (Fig. 3).

### In vitro activity and differentiation of osteoclasts from arthritic IL-33^-/-^ mice

**Fig. 4:**
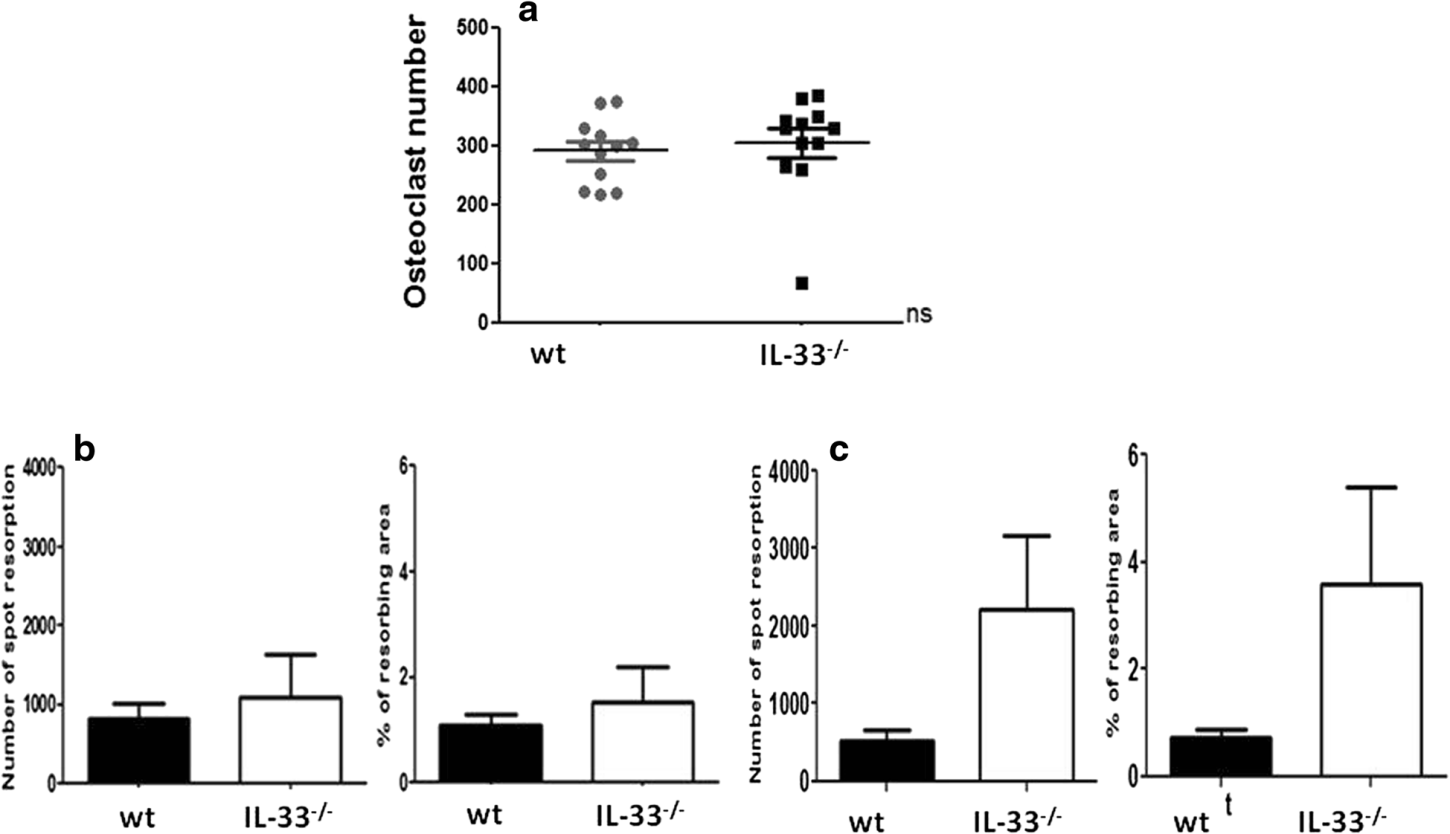
Osteoclastogenesis and bone resorption activity in WT and IL-33^-/-^ mice with collagen-induced arthritis (CIA). Mice as in Fig. 2 were evaluated for osteoclastogenesis (**a**) and bone resorption activity (**b**) in WT (n = 8, *black bars*) and IL-33^-/-^ (n = 8, *open bars*) mice at day 45 after CIA induction. Bone resorption was evaluated by number of spots with resorption and percentage resorption area. Arthritic mice (**c**) were defined as mice with a clinical score of arthritis >0 for at least 1 day during the CIA course (IL-33^-/-^, n = 6; WT n = 5). Data are mean ± SEM. *IL* interleukin, *WT* wild-type

IL-33 protects against bone resorption, but its role in osteoclastogenesis is unclear [25]. To examine whether IL-33 deficiency affects osteoclast formation during CIA, osteoclasts were generated from mouse bone marrow. The number of osteoclasts was similar for both IL-33^-/-^ and WT mice, so the absence of IL-33 did not affect in vitro differentiation of osteoclast precursors in the bone marrow of mice at day 45 of CIA (Fig. 4a). We then examined in vitro bone resorption activity of mouse osteoclasts and showed that number of resorption spots and percentage resorption area were lower, but not significantly, in WT than IL-33^-/-^ mice (Fig. 4b), especially when excluding mice without any arthritis from the analysis (Fig. 4c). Therefore, IL-33 might be involved in bone resorption during CIA.

### Psoriasis-like skin inflammation development is not impaired in IL-33^-/-^ mice

**Fig. 5:**
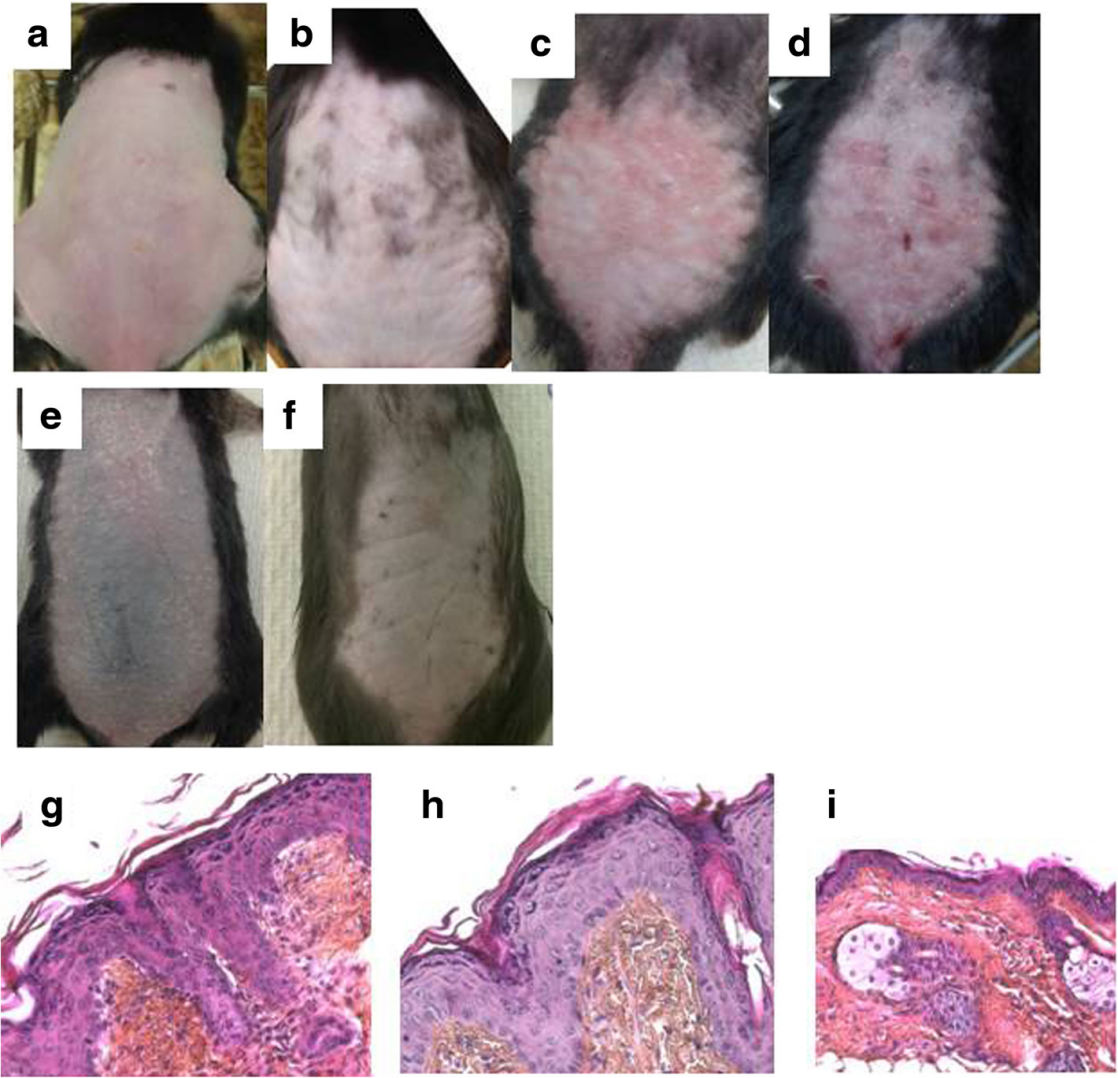
Clinical and histological evaluation of imiquimod (IMQ)-induced skin inflammation in mice. C57BL/6 mice, interleukin (IL)-33^-/-^ and wild-type (WT), were daily treated with IMQ cream or vehicle cream (Vaseline), applied on the shaved back skin. Phenotypical presentation of mouse back skin after shaving and before cream application showed a thin and white skin without scales (**a**). These characteristics were maintained 4 days after Vaseline application (**b**). In contrast, IMQ-treated mice showed erythema, infiltration, and scales, which appeared at day 4 in both IL-33^-/-^ (**c**) and WT (**d**) mice. Skin inflammation was increased similarly in both groups, with more scales and increased infiltration at day 9 in both IL-33^-/-^ (**e**) and WT (**f**) mice. Histological slides (×200) of back skin 11 days after IMQ application in IL-33^-/-^ mice (**g**) and WT mice (**h**) showed acanthosis and hyperkeratosis. Control WT mice without IMQ application is shown in (**i**)

**Fig. 6:**
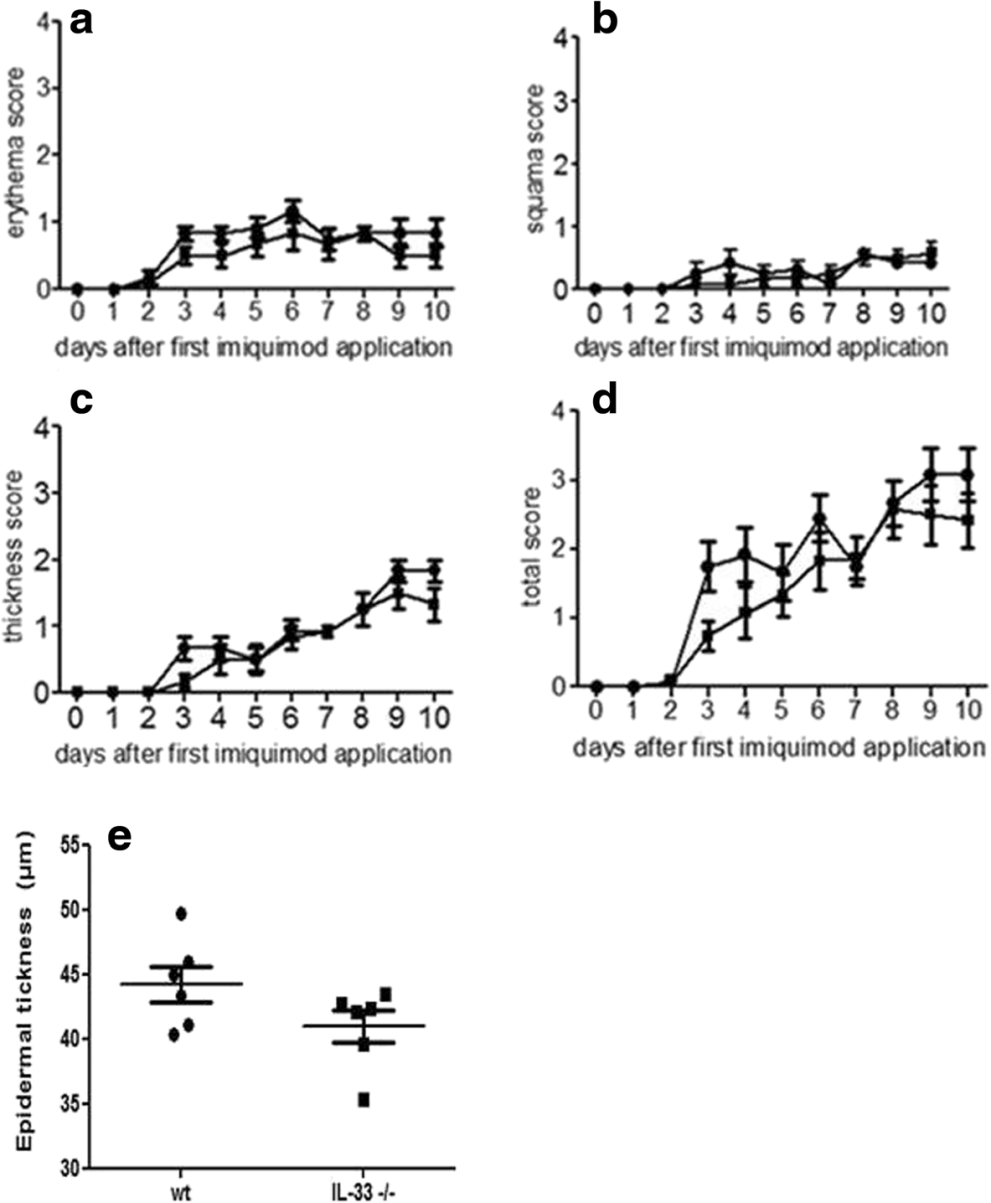
Imiquimod (IMQ)-induced skin inflammation develops independent of IL-33. IL-33^-/-^ (*n* = 6) or WT (*n* = 6) mice were treated daily with IMQ cream. Erythema, scales, and thickness of the back skin were scored daily on a scale from 0 to 4 (**a**-**c**). The cumulative score (erythema plus scales plus thickness) is depicted (**d**). Data are mean score ± SEM of six mice per group. Epidermal thickness was measured on histological slides of the skin at day 11 (**e**), and scores are individual scores for all mice (*n* = 6 in both groups). The results are representative of two experiments (*n* = 19). *IL* interleukin, *WT* wild-type

IL-33 is suggested to play a role in human skin psoriasis. This disease could also affect joints in psoriatic arthritis. Thus, we aimed to compare IMQ-induced psoriasis-like skin inflammation in WT and IL-33^-/-^ mice. At 2 days after the start of IMQ application for 10 days, the back skin of mice started to display signs of skin erythema and further thickness and scales from day 3. Typical examples of the psoriatic-like skin lesions are shown in Fig. 5 (c-f). Histological slides of the skin 11 days after the first application of IMQ are shown in Fig. 5 (g-i). Independent scores in a representative experiment are in Fig. 6 (a-c). From day 3 onward, the total inflammation score for severity continually increased, up to the end of the experiment. The total scores (thickness, scales and erythema) were similar in IL-33^-/-^ and WT mice (Fig. 6d). The two genotypes did not differ. Mean epidermal thickness was quantified on skin histological slides and was comparable between IL-33^-/-^ and WT mice (40.9 and 44.2 μm, *p* = 0.39, Student’s *t* test, Fig. 6e), in accordance with the clinical analysis.

**Fig. 7:**
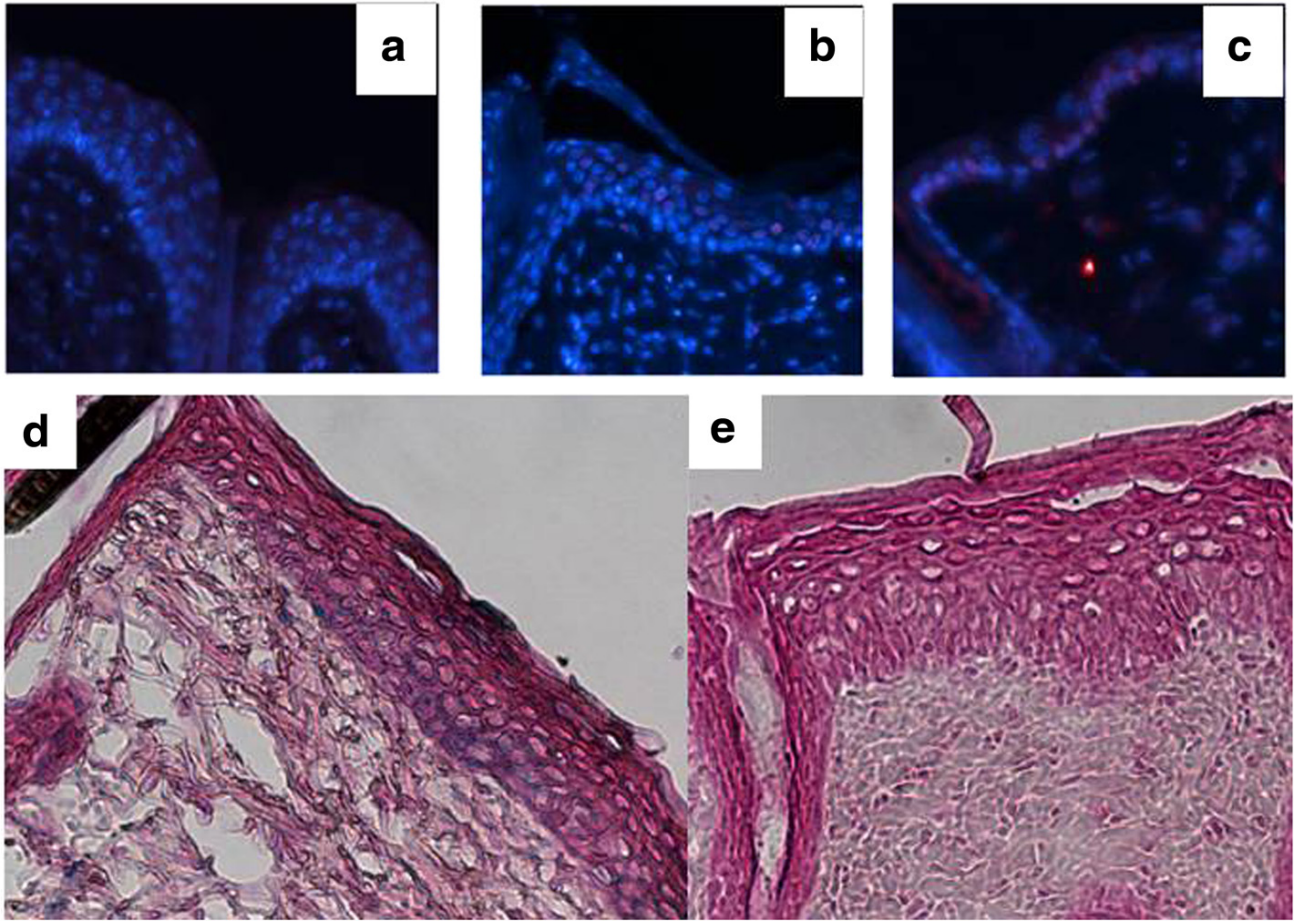
Interleukin (IL)-33 expression and promoter activation in the skin during imiquimod (IMQ)-induced skin inflammation. Immunofluorescence staining for IL-33 in skin samples 11 days after daily IMQ or Vaseline application. IMQ-treated IL-33^-/-^ mice showed no expression of IL-33 (**a**), whereas IMQ-treated wild-type (WT) mice showed IL-33 expression (*red*) in keratinocyte nuclei (**b**) as did WT mice without IMQ treatment (**c**). On the bottom panel, the activity of the endogenous IL-33 promoter, by X-Gal staining, showed IMQ-treated IL-33^-/-^ mice with expression in basal layers of the epidermis (**d**) and no detection in WT mice (**e**) (×200)

We found that the IL-33 promotor was active within the joints of mice during CIA, so we wondered whether IL-33 was expressed in the skin during IMQ-induced skin inflammation in mice. We first directly observed IL-33 expression by immunofluorescence staining of IL-33 in skin samples 11 days after daily application of IMQ. As expected, IL-33^-/-^ mice showed no expression of IL-33 (Fig. 7a), whereas WT mice with or without IMQ application showed nuclear expression of IL-33 in keratinocytes (Fig. 7b-c). We also evaluated IL-33 promoter activity by X-Gal staining in the skin of IL-33^-/-^ mice (Fig. 7d-e), which showed a similar pattern as in WT mice with IL-33 staining, namely lacZ staining within the basal layers of the epidermis (Figure D). As expected, no lacZ staining was detected in WT mice (Figure E).

## Discussion

In this study, we investigated the impact of IL-33 deficiency in two models of chronic inflammation, namely CIA in C57BL/6 mice and IMQ-induced psoriasis in mice. Arthritis and psoriasis developed independent of IL-33 expression, despite IL-33 promotor activation within the synovium during the course of arthritis and IL-33 expression in skin. Interestingly, IL-33 was detected in the synovium of RA patients [6] and in lesions of patients with psoriasis. Serum IL-33 levels are also increased in RA [5, 7] and psoriasis [26]. However, decreased epidermal IL-33 level could be rapid but transient, as demonstrated in experimentally induced psoriatic lesions in humans, and increased after 7 days [27]. Mice and humans showed strong differences in epidermal expression and regulation of IL-33, which could explain the inability of this model to reveal the pathophysiological role of IL-33 in psoriasis [28]. We cannot exclude that a compensation pathway of inflammation could be responsible for a lack of difference between WT and IL-33^-/-^ mice. A recent study showed that TLR7 dependence of IMQ-induced psoriatic lesions could be abolished only in MyD88-deficient mice and the inhibition of IL-1-alpha/beta or IL-1R1 was not sufficient [29].

The lack of IL-33 involvement in our model of CIA confirms findings from studies of other models of RA, namely antigen-induced arthritis and CIA [14] and K/BN serum transfer-induced arthritis [12]. In agreement with these observations, IL-33-deficient mice showed no difference from WT mice in experimental autoimmune encephalomyelitis, a model of multiple sclerosis, Con A-induced hepatitis, or streptozocyn-induced diabetes [30].

Conversely, IL-33 deficiency ameliorated dextran sodium sulfate-induced colitis and conferred resistance to lipopolysaccharide-induced endotoxinic shock in mice [29]. These two models involve innate immunity, whereas acquired immunity is involved in the above models of inflammation. IL-33 may be a major amplifier of systemic innate rather than acquired immunity.

Some studies of the K/BxN serum transfer model found reduced severity of arthritis in ST2-deficient mice [11]. These data suggest a crucial role of this receptor in the development of joint inflammation. This finding contrasts with all concordant studies of experimental chronic autoimmunity with IL-33-deficient mice. A similar contrast has been observed in other models such as OVA-induced airway inflammation [30]. One hypothesis to explain these apparent discrepancies is the existence of another ligand of ST2, even if to date, no study has revealed a potential candidate.

RA and psoriasis are chronic inflammatory diseases characterized by increased frequency of Th1 and Th17 cells. An increase in Th17 frequency and a decrease in Tregs frequency were observed in blood of patients with active RA [31]. Similarly, an increase in IL-17 level was observed in the skin of patients with psoriasis. Moreover, these patients showed a positive correlation between Th17/Treg cell ratio in the skin and disease activity, together with a decrease in the frequency and suppressive activity of Tregs [32], and anti-IL-17 antibody treatment conferred excellent therapeutic responses in patients with psoriasis [33].

We found no difference between IL-33^-/-^ and WT mice in frequencies of Treg, Th1, and Th17 cells in the spleen and lymph nodes with either CIA or psoriasis. Given the role of the cells in both models, this finding may explain why disease severity is not modified in the absence of IL-33.

RA is characterized by bone resorption and cartilage destruction. IL-33 is involved in bone resorption [34], and osteoblasts isolated from IL-33 transgenic mouse can produce significant bone matrix in vitro [34]. Additionally, the number of osteoclasts is decreased in these animals. ST2-deficient mice, with decreased bone density together with increased osteoclast number, showed an anti-osteoclastogenic effect of IL-33. Moreover, administration of IL-33 in human TNF-α transgenic mice, with spontaneous development of arthritis, inhibited bone destruction and reduced the number of osteoclasts [35]. Taken together, these studies show the inhibitory effect of IL-33 on osteoclasts and led us to evaluate osteoclastogenesis for the first time. Osteoclast formation was induced by culturing isolated bone marrow cells with RANKL and M-CSF for 5 days, followed by TRAP staining and revealed comparable osteoclastogenesis in IL-33^-/-^ and WT mice.

However, the resorption activity of a synthetic bone matrix by osteoclasts was higher in IL-33^-/-^ than WT mice. The differences were not statistically significant, probably because of the low number of animals used in this study. To our knowledge, IL-33 is not produced by osteoclasts. However, it might be produced by osteoclast precursors present at the initial periods of culture. As well, the osteoclast phenotype may be differentially regulated in vivo in IL-33^-/-^ mice and thus, the cells may respond to RANK-L and M-CSF in vitro stimulation. In addition, the effect of IL-33 on bone resorption may not be sufficient to change the clinical evolution of CIA. IL-33 may not have a direct role for osteoclasts but may exert its anti-osteoclastogenic activity via other cells such as osteoblasts [36].

## Conclusions

Even though IL-33 is expressed within the synovium of arthritic mice, CIA develops independently of IL-33, and in psoriasis, as well, its absence does not affect the T cell shift toward Th1, Th17, or Treg subpopulations. Altogether, these data suggest that this cytokine is not crucial for chronic inflammation development. Studies of RA patients are needed to determine whether treatment targeting the IL-33/ST2 axis would be effective.

## Abbreviations

CFA, complete Freund’s adjuvant; CIA, collagen-induced arthritis; CII, collagen type II; IFN, interferon; Ig, immunoglobulin; IL-, interleukin-; ILC2, type 2 innate lymphoid cells; IMQ, imiquimod; M-CSF, macrophage colony-stimulating factor; PBS, phosphate-buffered saline; RA, rheumatoid arthritis; RANKL, receptor activator of nuclear factor kappa B ligand; Th, T helper cells; TLR, Toll-like receptor; TNF, tumor necrosis factor; Treg, regulatory T cells, WT, wild-type
